# Impact of Personalised Feedback about Physical Activity on Change in Objectively Measured Physical Activity (the FAB Study): A Randomised Controlled Trial

**DOI:** 10.1371/journal.pone.0075398

**Published:** 2013-09-16

**Authors:** Job G. Godino, Clare Watkinson, Kirsten Corder, Theresa M. Marteau, Stephen Sutton, Stephen J. Sharp, Simon J. Griffin, Esther M. F. van Sluijs

**Affiliations:** 1 MRC Epidemiology Unit, University of Cambridge, Institute of Metabolic Science, Addenbrooke’s Hospital, Cambridge, United Kingdom; 2 UKCRC Centre for Diet and Activity Research (CEDAR), Institute of Public Health, University of Cambridge, Cambridge, United Kingdom; 3 Behaviour and Health Research Unit, Institute of Public Health, University of Cambridge, Cambridge, United Kingdom; 4 Behavioural Science Group, Institute of Public Health, University of Cambridge, Cambridge, United Kingdom; Iran University of Medical Sciences, Islamic Republic Of Iran

## Abstract

**Background:**

Low levels of physical activity are a major public health concern, and interventions to promote physical activity have had limited success. Whether or not personalised feedback about physical activity following objective measurement motivates behaviour change has yet to be rigorously examined.

**Methods:**

**And Findings**: In a parallel group, open randomised controlled trial, 466 healthy adults aged 32 to 54 years were recruited from the ongoing population-based Fenland Study (Cambridgeshire, UK). Participants were randomised to receive either no feedback until the end of the trial (control group, n=120) or one of three different types of feedback: *simple*, *visual*, or *contextualised* (intervention groups, n=346). The primary outcome was physical activity (physical activity energy expenditure (PAEE) in kJ/kg/day and average body acceleration (ACC) in m/s^2^) measured objectively using a combined heart rate monitor and accelerometer (Actiheart^®^). The main secondary outcomes included self-reported physical activity, intention to increase physical activity, and awareness of physical activity (the agreement between self-rated and objectively measured physical activity). At 8 weeks, 391 (83.9%) participants had complete physical activity data. The intervention had no effect on objectively measured physical activity (PAEE: β=-0.92, 95% CI=-3.50 to 1.66, *p*=0.48 and ACC: β=0.01, 95% CI=-0.00 to 0.02, *p*=0.21), self-reported physical activity (β=-0.39, 95% CI=-1.59 to 0.81), or intention to increase physical activity (β=-0.05, 95% CI=-0.22 to 0.11). However, it was associated with an increase in awareness of physical activity (OR=1.74, 95% CI=1.05 to 2.89). Results did not differ according to the type of feedback.

**Conclusions:**

Personalised feedback about physical activity following objective measurement increased awareness but did not result in changes in physical activity in the short term. Measurement and feedback may have a role in promoting behaviour change but are ineffective on their own.

**Trial Registration:**

Current Controlled Trials ISRCTN92551397 http://www.controlled-trials.com/ISRCTN92551397

## Introduction

Low levels of physical activity are independently associated with increased risk of mortality, obesity, type 2 diabetes, cardiovascular disease, and some cancers [1,2]. In recent years, government sponsored programs have been developed to inform the public about the health benefits of regular physical activity [3,4], and family doctors in the UK and elsewhere have been called on to assess and address their patients’ physical inactivity [5]. In spite of these efforts, only 34% of UK adults report currently achieving the recommendation of 30 minutes of moderate to vigorous physical activity per day on at least 5 days of the week. Only 5% achieve this level when physical activity is measured by accelerometer [6].

To date, interventions implemented in primary care and community settings to promote physical activity have had limited success. When effects have been observed, they have tended to be small and short-term [7-10]. This may be due in part to the fact that many sedentary individuals are unaware that their current physical activity level is inadequate [11-14], and hence they may not perceive a need to change their behaviour. Previous studies have reported that up to 61% of inactive adults overestimated the amount of activity that they engage in [11-14]. Importantly, overestimators were less likely to report an intention to increase their physical activity than individuals who more accurately reported their inactivity [12,13].

Evidence suggests that personalised feedback about physical activity following objective measurement may increase awareness of physical activity [15,16]. This in turn may stimulate intention to increase physical activity, which ultimately may lead to positive changes in behaviour [17-20]. This pathway is consistent with several health behaviour theories, including the theory of planned behavior [21-23], and may be part of the mechanism underlying the apparent effectiveness of pedometers [24]. However, the evidence is limited by studies with small sample sizes and imprecise self-reported outcome measures.

It is also possible that personalised feedback about physical activity could have harmful consequences [18,25]. This means that results indicating high levels of physical activity might generate false reassurance and a subsequent reduction in activity levels. In contrast, results indicating low levels of physical activity might trigger denial or fatalistic attitudes leading to no increase in physical activity and perhaps even a decline [18]. There appears to be no research available addressing these questions in the context of physical activity promotion.

Despite its potential as a tool for promoting behaviour change, the independent effects of personalised feedback about physical activity on behaviour have yet to be rigorously evaluated using a precise objective measure of physical activity. We assessed whether feedback influences objectively measured physical activity, self-reported physical activity, intention to increase physical activity, awareness of physical activity, and other theory-based cognitive and emotional antecedents to health behaviour change in a population-based sample of UK adults. Additionally, we aimed to test whether the observed effects differed according to three different types of feedback that contained visual images and goal setting by way of behavioral modelling, each of which has evidence of effectively motivating behaviour change [26-29].

## Methods

### Ethics Statement

The protocol for this trial and supporting CONSORT checklist are available as supporting information (see Checklist S1 and Protocol S1). The study methods have been described in detail elsewhere [30].

Full ethical approval was obtained from the Cambridgeshire 2 Research Ethics Committee on May 3, 2007 (07/Q0108/79). Written informed consent was obtained from each participant. The study is registered with Current Controlled Trials: ISRCTN92551397 (http://www.controlled-trials.com/ISRCTN92551397).

### Study Design

The Feedback, Awareness and Behaviour (FAB) study was a parallel group, open randomised controlled trial in which eligible participants were allocated to receive either no personalised feedback about physical activity (control group), or one of three different types of feedback: *simple*, *visual*, or *contextualised* (intervention groups).

### Recruitment

Participants were recruited from the Fenland Study, an ongoing population-based, observational study among residents of Cambridgeshire born between 1950 and 1975 investigating the influence of lifestyle and genetic factors on the development of diabetes, obesity, and other metabolic disorders [31]. Exclusion criteria assessed by family doctors included diabetes, a terminal illness with a prognosis of less than one year, psychotic illness, pregnancy or lactation, and inability to walk unaided. Approximately 28% of those registered with participating general practices are enrolled in the Fenland Study. Between September 2007 and August 2008, all participants were invited to take part in the FAB study through a letter which explained that the aim of the study was ‘to investigate the effects of the Fenland Study experience on participants and to help us understand the best way of providing people with feedback on their health’.

### Baseline Assessment

Potential participants completed questionnaires including psychological outcome measures, demographic characteristics, medical history, general lifestyle, and physical activity. They underwent anthropometric, body composition, clinical, and fitness measurements, and blood samples were collected, all by trained staff following standard operating procedures. Finally, they were fitted with a combined heart rate monitor and accelerometer (Actiheart^®^) [32], which they were instructed to wear continuously for six days and nights prior to returning it to the study centre. Potential participants were excluded from the FAB study if they developed a rash while wearing the Actiheart^®^ and/or it recorded less than 36 hours of data.

### Randomisation, Allocation Concealment, and Blinding

Following the download of Actiheart^®^ data, participants were randomly allocated to one of the study groups by a minimisation program implemented independently by a statistician. Minimisation was based on sex and the median values from the first 1000 Fenland Study participants for age (<45 and >45 years), BMI (<27 and >27 kg/m^2^), HbA_1c_ (<35.5 and >35.5 mmol/mol), and physical activity level (PAL: <1.63, >1.63). PAL was calculated as the ratio of total energy expenditure in a 24-hour period to basal metabolic rate. Allocation was concealed from the study coordination team, researchers, and participants until the interventions were assigned. It was not possible to blind participants to whether or not they were allocated to the control group, but participants allocated to one of the intervention groups were unaware of the specific details of each type of feedback. Furthermore, researchers who assessed the baseline characteristics of participants and the primary outcome of the trial remained blinded to group assignment. Additionally, an independent, quality assured data-entry company undertook all data entry unaware of group allocation.

### Intervention

The interventions consisted of three different types of personalised feedback about physical activity sent by post, along with an instruction letter and a brief questionnaire, as soon as possible following the download of Actiheart^®^ data and randomisation of participants. The instruction letter asked participants in the intervention groups to read through their feedback until they understood it and prior to completing and returning the questionnaire. A reminder letter, along with a second copy of the questionnaire, was sent if responses were not received within two weeks. Control group participants only received an instruction letter and a brief questionnaire, and they did not receive any feedback until after they completed follow-up. In order to isolate the effects of their individual components, each type of feedback built upon the previous with *contextualised* hypothesized to have the largest effect, followed by *visual* and then *simple*.

*Simple* feedback consisted of a short definition of physical activity, a summary of the health benefits of physical activity, and a brief description of current physical activity guidelines. It also included the participant’s average PAL, which was calculated using the Actiheart^®^ software and presented alongside of a table showing the five FAO/WHO/UNU reference categories for PAL, ranging from <1.2 (bed-rested) to >1.95 (very high activity level) [33].

There is evidence that feedback containing personalised visual images may be more effective at eliciting behaviour change than written or verbal feedback alone [26,27]. Thus, the *visual* feedback consisted of the *simple* feedback, supplemented by a series of line graphs that displayed the participant’s heart rate and daily movement counts over each 24-hour period that they wore the monitor.

There is evidence that goal setting and behavioural modelling are associated with positive behaviour change [29]. On this basis, *contextualised* feedback consisted of the *simple* and *visual* feedback, supplemented by estimates of the added PAL value of familiar activities (e.g., housework, walking, or cycling) calculated for various durations so that participants could establish goals to increase their activity [34]. Goal setting was modelled with a short, gender-specific vignette based on a ‘typical’ Fenland Study participant.

The interventions were pilot tested with twenty Fenland Study participants who were asked to read through example feedback prior to taking part in a short structured interview. This aimed to explore their understanding, attitude, and preferences in relation to the information presented, and minor revisions were made on the basis of the results. Example feedback was published as a supplement to the study protocol, which is freely available [30].

### Follow-up Assessment

Approximately eight weeks after randomisation, participants were sent a questionnaire and an Actiheart^®^ which they wore for another period of six days and nights continuously.

### Measurements

#### Primary outcome

The primary outcome was objectively measured physical activity. It was assessed at baseline and at follow-up using the Actiheart^®^, a combined heart rate monitor and accelerometer. It is non-invasive, weighs less than 8 grams, and is worn on the chest attached to standard electrocardiogram electrodes that are stuck directly onto the skin. It is only 7 mm thick (33 mm in diameter), and except for a brief period to change electrodes (once every few days), it does not need to be removed. The device is also waterproof and can be worn while swimming or showering. These factors make it discreet and convenient to wear. Physical activity was defined as physical activity energy expenditure (PAEE, measured in kJ/kg/day) and average body acceleration (ACC, measured in m/s^2^). Branched equation modelling was utilised to estimate PAEE from acceleration and heart rate data [35]. This approach has high validity and reliability for estimating the volume and intensity of physical activity [36,37] and overcomes some of the key limitations associated with either accelerometers or heart rate monitors alone [32].

#### Secondary outcomes

Secondary outcomes included self-reported physical activity (measured via the previously validated Recent Physical Activity Questionnaire in MET h/day [38]), intention to increase physical activity, awareness of physical activity, and other theory-based cognitive and emotional antecedents to behaviour change. Awareness was assessed with two self-rated measures of physical activity. Participants were asked which of the five reference categories they believed best described their PAL (reference standard) and whether or not they had engaged in the recommended amount of physical activity over the preceding month (recommendation standard: a PAL > 1.7 was considered equivalent to meeting the recommendation). They were then categorised as aware or unaware according to the concordance between their self-rated and objectively measured physical activity [11-14]. The hypothesized cognitive and emotional antecedents to behaviour change were drawn from the previously validated ProActive study questionnaires, which were largely based on the theory of planned behavior [21], and were amended according to guidelines where appropriate [39,40]. They were measured using 5-point Likert scales ranging from 1 (‘strongly disagree’) to 5 (‘strongly agree’) with higher scores indicating a greater likelihood of behaviour change. Worry and concern about physical activity over the preceding two weeks were measured using similar scales ranging from 1 (‘not at all’) to 5 (‘almost all of the time’). Time orientation was measured at baseline using a nine-item form of the previously validated Zimbardo Time Perspective Inventory [41]. Acceptability was assessed at follow-up using six items intended to gauge if the interventions were understood and well received by participants. Medical and psychological adverse events were monitored throughout the trial.

### Sample Size

In the ProActive trial, which had a similar sample population and primary outcome to the FAB study [42], the mean (standard deviation) PAEE at baseline was 0.116 (0.076) kJ/kgFFM/min, and the correlation between baseline and follow-up PAEE was 0.58. In order to detect a difference between groups of 0.025 kJ/kgFFM/min in PAEE (which equates to roughly 20 minutes of brisk walking per day) at follow-up, with a significance level of 5% and 80% statistical power, approximately 400 participants were required to complete the trial. To allow for 20% loss to follow-up, we aimed to randomise 500 participants. To ensure adequate power for all comparisons, these analyses assumed equal-sized groups (an intervention group to control group ratio of 1:1).

### Statistical Analysis

Analyses to estimate intervention effects compared values at follow-up, adjusted for baseline, between randomised groups. Analysis of covariance (ANCOVA) was used to compare objectively measured physical activity between those who received feedback (intervention groups) and those who did not (control group). The assumptions of ANCOVA were tested and met.

Similar ANCOVA procedures were used to examine differences in self-reported physical activity, intention to increase physical activity, and other cognitive and emotional theory-based antecedents to health behaviour change. Logistic regression was used to examine differences in awareness of physical activity. Only participants with complete outcome data were included in the analyses (a complete case analysis). Those with missing baseline data were included using the missing-indicator method. To investigate the effect of having excluded participants with missing data, a sensitivity analysis that utilised a multiple imputation procedure with a ‘missing at random’ assumption was undertaken on the primary outcome.

Additional pre-planned analyses were conducted. The presence of a dose-response was examined by means of single degree of freedom orthogonal contrasts. The c*ontextualised* and *visual* feedback was compared with *simple* feedback, and *contextualised* feedback with *visual* feedback. Subgroup analyses were conducted to determine if sex (male and female), baseline PAEE, PAL (<1.7 and > 1.7), and awareness of physical activity (aware and unaware) moderated intervention effects on physical activity. Time orientation was also examined as a potential moderator of the intervention effect in a post-hoc analysis. All analyses were performed on an intention-to-treat basis (analysis of data according to randomised study group) using STATA software [43].

## Results

### Participants

**Figure 1:**
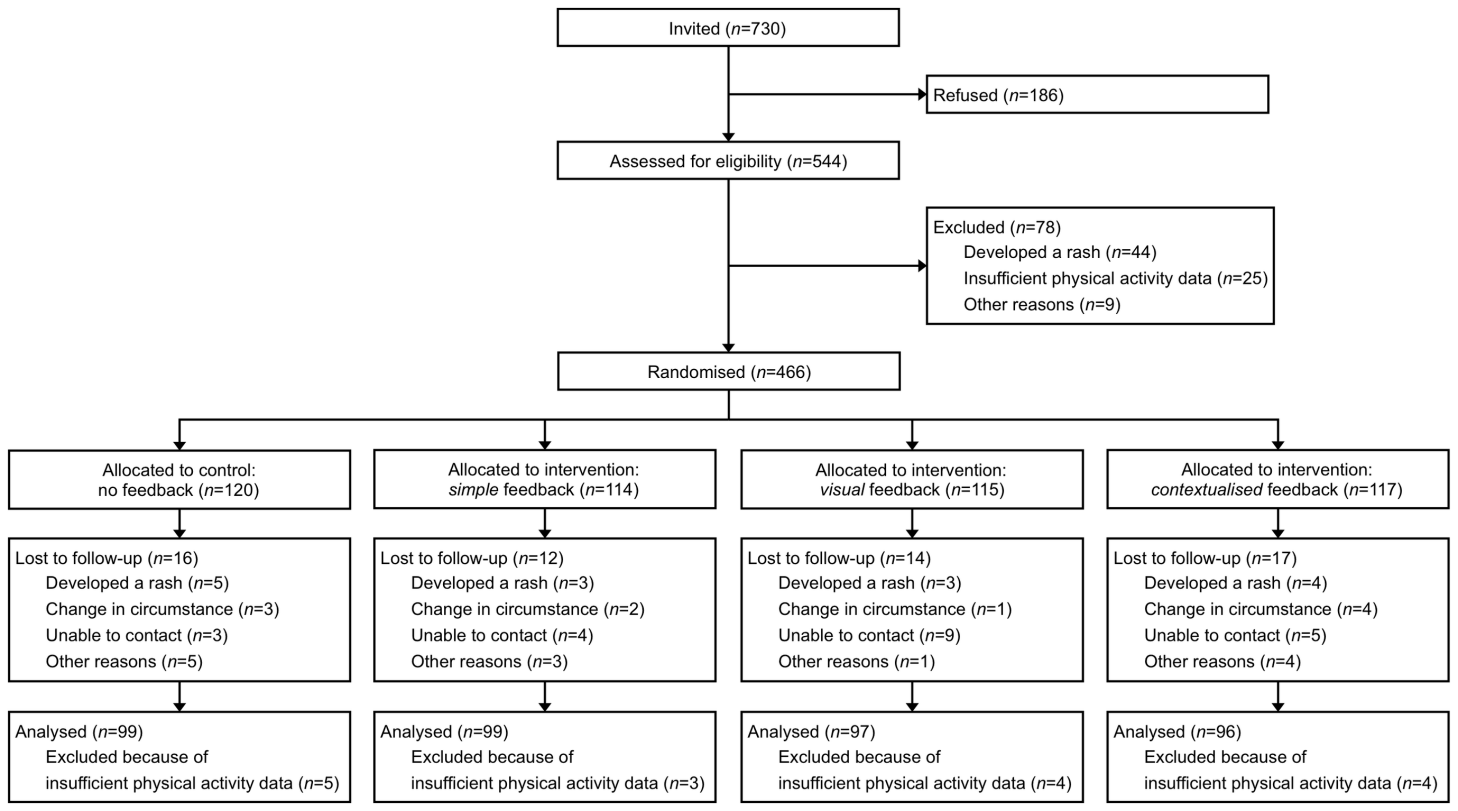
Flow of participants through the Feedback, Awareness and Behaviour study.

Figure 1 shows the flow of participants through the trial. Between September 2007 and August 2008, 730 Fenland Study participants were invited to take part in the FAB study and 544 were assessed for eligibility. Those who refused did not differ from those who were assessed for eligibility according to age, sex, BMI, HbA_1c_ level or PAL. A total of 466 met the inclusion criteria and were subsequently randomised: 346 were allocated to receive personalised feedback about physical activity (intervention groups) and 120 were allocated to receive no feedback (control group). According to our knowledge, all randomised participants received the interventions as allocated. Potential participants who did not meet the inclusion criteria did not differ from those who were randomised according to age, sex, BMI, HbA_1c_ level or PAL.

Baseline characteristics of participants did not differ between groups as shown in Table 1. They had a mean (SD) age of 47.5 (6.8) years, and the majority were female (53.4%) and white (98.3%). The mean (SD) age at which they finished full-time education was 18.0 (3.6) years and most were employed full-time (66.5%). Most participants rated their health as good, very good, or excellent (73.4%) although the majority were overweight (mean (SD) BMI of 27.5 (5.2) kg/m^2^) and relatively inactive (mean (SD) PAL of 1.71 (0.26)). Their HbA_1C_ level was in the normal range (mean (SD) HbA_1C_ of 35.8 (5.4) mmol/mol), 14.7% were current smokers, and 26.8% consumed more than 10 units of alcohol per week.

**Table 1:** Participant demographic characteristics and health risk factors at baseline by allocation to receive either no physical activity feedback (control group) or to receive *simple*, *visual*, or *contextualised* physical activity feedback (intervention groups) (*n*=466).

	**Control (*n*=120**)	***Simple* (*n*=114**)	***Visual* (*n*=115**)	***Contextualised* (*n*=117**)
Age (years)	47.9 (7.0)	47.5 (6.9)	47.0 (6.6)	47.7 (6.5)
Men, No. (%)	57 (47.5)	51 (44.7)	54 (47.0)	55 (47.0)
White ethnicity, No. (%)	118 (98.3)	110 (96.5)	114 (99.1)	116 (99.2)
Age finished full-time education (years)	18.0 (3.6)	18.2 (3.9)	17.7 (3.1)	18.1 (3.7)
Employed full-time, No. (%)	77 (64.2)	86 (75.4)	71 (61.7)	76 (65.0)
Current smoker, No. (%)	23 (19.7)	15 (13.6)	12 (10.5)	17 (14.7)
Consume more than 10 units of alcohol per week, No. (%)	30 (25.0)	31 (27.2)	30 (26.1)	34 (29.1)
Self-rated health good, very good, or excellent, No. (%)	87 (72.5)	85 (74.6)	85 (73.9)	85 (72.7)
Body mass index (kg/m^2^)	27.3 (4.9)	27.9 (5.0)	27.3 (5.8)	27.5 (4.9)
HbA_1C_ (mmol/mol)	35.6 (3.9)	36.0 (4.0)	34.9 (3.9)	36.8 (8.4)
Physical activity level (ratio of total energy expenditure in a 24-hour period to basal metabolic rate)	1.72 (0.27)	1.71 (0.26)	1.70 (0.25)	1.69 (0.25)

Values are means (standard deviations) unless otherwise specified.

### Outcomes

#### Primary outcome

391 (83.9%) participants had complete physical activity data, 59 (12.7%) were lost to follow-up, and 16 (3.4%) were excluded from the primary analyses because insufficient data were recorded. Participants excluded from the primary analyses had a greater BMI than those analysed (*p* = 0.03) but were otherwise similar. Mean (SD) duration of follow-up was 8.1 (3.4) weeks with no difference between groups. There was no difference in objectively measured physical activity between those who did and did not receive feedback as shown in Table 2. Results were unaffected by including imputed data at follow-up. There was no difference between groups according to the type of feedback and no evidence that effects differed by sex, baseline PAEE, PAL, awareness of physical activity, or time orientation.

**Table 2:** Intervention effects on physical activity: combined intervention groups versus control group (*n*=391).

	**Control (n=99**)		***Simple* (n=99**)		***Visual* (n=97**)		***Contextualised* (n=96**)		Adjusted Intervention Effect*	
	Baseline	Follow-up	Baseline	Follow-up	Baseline	Follow-up	Baseline	Follow-up	Coefficient (95% CI)	*p*-value
Physical activity energy expenditure (kJ/kg/day)	47.4 (15.4)	48.0 (17.4)	45.7 (15.3)	46.9 (14.7)	45.8 (17.0)	45.4 (15.4)	44.7 (14.0)	44.5 (13.7)	-0.92 (-3.50 to 1.66)	0.48
Average body acceleration (m/s^2^)	0.11 (0.05)	0.11 (0.05)	0.12 (0.04)	0.12 (0.04)	0.12 (0.08)	0.12 (0.06)	0.12 (0.05)	0.12 (0.05)	0.01 (-0.00 to 0.02)	0.21

Values are means (standard deviations). * Adjusted intervention effect refers to the difference in means between the combined intervention groups and the control group, adjusted for baseline.

#### Secondary outcomes

Compared to those in the control group, participants who received feedback had greater awareness of physical activity, as shown in Table 3. More specifically, the odds of a participant in the intervention groups being classified as aware in relation to the reference and recommendation standards were 1.76 and 1.74 times greater than the odds of those in the control group. There were no differences in self-reported physical activity, intention to increase physical activity, perceived adequacy, behavioural beliefs, self-efficacy, perceived importance and worry or concern about physical activity between the feedback and control groups as shown in Table 4. Participants who received feedback did report increased subjective norm, but the effect size was small (0.21). The different types of feedback did not independently affect any of the aforementioned secondary outcomes.

**Table 3:** Intervention effects on awareness of physical activity levels: combined intervention groups versus control group (*n*=381).

	**Control (*n*=99**)		***Simple* (*n*=97**)		***Visual* (*n*=93**)		***Contextualised* (*n*=92**)		Adjusted Intervention Effect*
	Baseline	Follow-up	Baseline	Follow-up	Baseline	Follow-up	Baseline	Follow-up	Odds Ratio (95% CI)
Awareness: reference standard†	32 (32.3)	28 (28.3)	17 (17.5)	29 (29.9)	24 (25.8)	39 (41.9)	23 (25.0)	39 (42.4)	1.76 (1.05 to 2.96)
Awareness: recommended standard‡	49 (49.5)	46 (46.5)	60 (62.5)	63 (65.6)**	47 (51.1)	54 (58.7)**	52 (57.1)	53 (58.2)**	1.74 (1.05 to 2.89)

Values are numbers (percentages). * Adjusted intervention effect refers to the odds ratio comparing the combined intervention groups with the control group, adjusted for baseline. ** Missing one data point. †Participants were asked which of the five FAO/WHO/UNU reference categories they believed best described their physical activity level and ‡ whether or not they had engaged in the recommended amount of physical activity over the preceding month (a PAL > 1.7 was considered equivalent to meeting the recommendation). They were then categorised as aware or unaware according to the concordance between their self-rated and objectively measured physical activity.

**Table 4:** Intervention effects on cognitive and emotional factors: combined intervention groups versus control group (*n*=407).

	**Control (*n*=104**)		***Simple* (*n*=102**)		***Visual* (*n*=101**)		***Contextualised* (*n*=100**)		Adjusted Intervention Effect*
	Baseline	Follow-up	Baseline	Follow-up	Baseline	Follow-up	Baseline	Follow-up	Coefficient (95% CI)
Self-reported physical activity (MET h/day)	10.84 (7.70)	10.55 (7.77)	13.21 (8.04)	12.08 (7.76)	10.75 (8.17)	10.53 (8.84)	10.83 (7.87)	9.78 (6.62)	-0.39 (-1.59 to 0.81)
Intention to increase physical activity	3.31 (0.90)	3.36 (0.71)	3.34 (0.88)	3.38 (0.78)	3.27 (0.80)	3.16 (0.91)	3.39 (0.91)	3.40 (0.75)	-0.05 (-0.22 to 0.11)
Perceived adequacy	3.10 (1.06)	3.33 (0.97)	3.26 (1.03)	3.34 (0.87)	3.22 (0.96)	3.27 (1.01)**	3.14 (1.04)	3.24 (0.97)	-0.13 (-0.30 to 0.05)
Behavioral beliefs	3.86 (0.59)	3.74 (0.62)	3.82 (0.76)	3.69 (0.63)	3.75 (0.67)	3.68 (0.81)	3.83 (0.56)	3.66 (0.58)	-0.00 (-0.13 to 0.12)
Subjective norm	3.15 (0.98)	3.04 (0.81)	3.14 (1.04)	3.18 (0.94)	3.03 (0.99)	3.14 (1.05)	3.10 (0.98)	3.14 (0.90)	0.20 (0.04 to 0.36)
Self-efficacy	3.65 (0.78)	3.57 (0.73)	3.73 (0.70)	3.49 (0.80)	3.74 (0.69)	3.59 (0.76)	3.69 (0.83)	3.56 (0.79)	-0.06 (-0.22 to 0.09)
Perceived importance	4.50 (0.52)	4.38 (0.70)	4.56 (0.58)	4.37 (0.58)	4.50 (0.52)	4.35 (0.62)	4.50 (0.50)	4.23 (0.72)	-0.06 (-0.20 to 0.08)
Worry	2.60 (0.92)	2.44 (0.85)	3.01 (0.82)	2.83 (0.86)	2.70 (0.87)	2.64 (0.84)	2.86 (0.91)	2.60 (0.83)	0.11 (-0.04 to 0.26)
Concern	2.38 (1.01)	1.99 (0.89)	2.53 (0.91)	2.12 (0.89)	2.41 (0.90)	2.09 (0.95)	2.45 (0.91)	2.18 (0.88)	0.13 (-0.04 to 0.31)

Values are means (standard deviations). * Adjusted intervention effect refers to the difference in means between the combined intervention groups with the control group, adjusted for baseline. ** Missing two data points.

#### Acceptability and adverse events

Among those who received feedback, the majority (86.1%) reported that they were either fairly or very confident that they understood it. Most participants felt fairly or very confident that their feedback was accurate (78.7%), that it was clear and easy to understand (84.7%), and that it applied to them personally (77.6%). The vast majority of participants (93.2%) kept their feedback and many (77.0%) discussed it with others (e.g., family members, friends, or health professionals). No serious adverse events related to the interventions were reported.

## Discussion

Among middle-aged men and women who underwent objective measurement of physical activity, the provision of personalised feedback about physical activity was not associated with changes in physical activity after eight weeks. These results were not influenced by the type of feedback provided, sex, or baseline physical activity (PAEE or PAL), awareness of physical activity, or time orientation. Previous research highlights the importance of examining the potential negative effects of feedback on behaviour [18,25], although the evidence in the context of physical activity promotion is limited. We found no evidence that participants who received feedback indicating that they were sufficiently active were falsely reassured. Conversely, feedback suggesting that participants were not sufficiently active did not appear to trigger denial or fatalistic attitudes.

After eight weeks, participants reported that they understood their feedback, discussed it with others and were confident that it was accurate. Feedback was associated with an increase in awareness of physical activity such that those in the intervention groups, all of whom received a numeric PAL value at baseline, were more likely than those in the control group to accurately report their PAL and whether or not they were meeting the national physical activity guidelines. Given that the feedback did not explicitly state whether or not a participant was meeting the guidelines, this effect may have been the result of a heightened consciousness about physical activity in general. Nevertheless, with the exception of subjective norms, feedback about physical activity was not associated with changes in any of the other cognitive and emotional antecedents to behaviour change.

Taken together, these findings suggest that although feedback may moderately increase awareness of behaviour, this is not by itself sufficient to motivate or cue changes in behaviour in the short-term, although it is possible that it might enhance the effects of a more intensive behaviour change intervention. The absence of an effect on behaviour may be due to the use of a single occasion of measurement and feedback. A higher frequency of measurement and feedback might have elicited a greater behavioural response. Studies of self-monitoring, which might be viewed as a form of repeated or continuous measurement and feedback, highlight the potential of such an approach. Indeed, a recent meta-analysis of trials of pedometer interventions showed that feedback on the number of steps a person takes results in moderate increases in physical activity [24]. However, in contrast to the current study, none of the included trials incorporated measures of overall physical activity energy expenditure into their design.

### Strengths and Limitations of the Study

This trial has several important strengths. We recruited a relatively large population-based sample with a low refusal rate (25.4%). The characteristics of those who agreed and declined to participate were similar, suggesting that our results are likely to be generalisable to the Fenland Study population at large. Study groups were well matched at baseline. We used a precise objective measure of physical activity (combined heart rate monitor and accelerometer) that has greater validity and reliability than commonly used self-report questionnaires and is more accurate than either accelerometers or heart-rate monitors alone [32]. Those undertaking data entry and assessing the primary outcome were unaware of participants’ group allocation. We tested three different types of feedback, each containing elements previously incorporated into effective behaviour change interventions. Participant retention in the trial was high (87.3%) and did not differ between study groups.

Limitations include our use of the Actiheart^®^ software, which meant that we could only provide feedback in terms of PAL rather than the number of minutes per day spent in moderate to vigorous physical activity as describe in the national guidelines. Although the majority of those who received feedback reported that they understood it, it is possible that feedback reflecting the national guidelines that many individuals are familiar with might have been viewed as more salient and might have promoted positive changes in physical activity. Additionally, it is possible that measurement without feedback might promote change in behaviour, leading to a potential ceiling effect or a level of effect that is difficult to increase with a minimal intervention. This might explain why we did not observe significant differences in physical activity between the control group and intervention groups [44]. However, this is something that we were unable to test in this trial. Finally, nearly all participants were from one location in the UK, from one ethnic group, and were physically and psychologically healthy. Consequently, the results might not generalise to other settings or groups, such as those with greater disease risk and hence potentially more to gain from increasing their physical activity.

### Future Research

Advances in technology will make it ever cheaper and easier to measure the physical activity of large numbers of individuals and provide them with instantaneous, personalised feedback. Indeed, there are many efforts to use the rapidly expanding world of smart phones and tablet computers to accomplish this [45,46]. In addition, inexpensive consumer devices have been developed specifically to measure a variety of behaviours, including physical activity, diet, and sleep. These devices have been integrated with computer technology so that users are provided with highly sophisticated personalised feedback. While such tools could be used by family doctors in primary care to assess their patients’ physical activity, our data suggest that although they may increase awareness of behaviour, they are unlikely to effectively promote behaviour change on their own. More research is needed to understand how best to utilise new technologies to promote uptake and maintenance of healthy behaviours, and to determine whether the incorporation of personalised feedback into a more intensive behaviour change intervention might enhance its efficacy. A greater understanding of the wider collective determinants of physical activity is also necessary to inform population-level interventions addressing economic, environmental, and sociocultural barriers to behaviour change [47]. In the meantime, doctors wishing to help their patients to increase their physical activity might recommend use of pedometers.

## Conclusions

The provision of personalised feedback about physical activity did not increase physical activity more than measurement of physical activity alone. However, feedback was associated with increases in awareness of physical activity. These results suggest that when promoting physical activity, increasing awareness may be necessary but insufficient, and simply providing individuals with feedback about their behaviour is unlikely to facilitate behaviour change.

## Supporting Information

Checklist S1 **CONSORT Checklist.**
(DOC)

Protocol S1 **Trial Protocol.**
(PDF)

## References

[1] Lee IM, Shiroma EJ, Lobelo F, Puska P, Blair SN et al. (2012) Effect of physical inactivity on major non-communicable diseases worldwide: an analysis of burden of disease and life expectancy. Lancet 380: 219-229. doi:10.1016/S0140-6736(12)61031-9. PubMed: 22818936. 22818936 10.1016/S0140-6736(12)61031-9 PMC3645500

[2] Department of Health (2004) Physical Activity, Health Improvement and Prevention At least five a week: Evidence on the impact of physical activity and its relationship to health. London: Department of Health.

[3] Department of Health (2011) Change4Life: Move more. London: Department of Health Available: http://www.nhs.uk/Change4Life/Pages/be-more-active.aspx. Accessed 08 August 2013.

[4] Department of Health (2011) NHS Choices: Live Well. London: Department of Health Available: http://www.nhs.uk/livewell/Pages/Livewellhub.aspx. Accessed 08 August 2013.

[5] Khan KM, Weiler R, Blair SN (2011) Prescribing exercise in primary care. BMJ 343: d4141. doi:10.1136/bmj.d4141. PubMed: 21765112. 21765112 10.1136/bmj.d4141

[6] Craig R, Mindell J, Hirani V (2009) Health Survey for England 2008 Volume 1: Physical activity and fitness. London: The NHS Information Centre for Health and Social Care.

[7] van Sluijs EM, van Poppel MN, van Mechelen W (2004) Stage-based lifestyle interventions in primary care: are they effective? Am J Prev Med 26: 330-343. doi:10.1016/j.amepre.2003.12.010. PubMed: 15110061. 15110061 10.1016/j.amepre.2003.12.010

[8] Hillsdon M, Foster C, Thorogood M (2005) Interventions for promoting physical activity. Cochrane Database Syst Rev: CD003180. doi:10.1002/14651858.CD003180.pub2. PubMed: 15674903. 10.1002/14651858.CD003180.pub2 PMC4164373 15674903

[9] Müller-Riemenschneider F, Reinhold T, Nocon M, Willich SN (2008) Long-term effectiveness of interventions promoting physical activity: A systematic review. Prev Med 47: 354-368. doi:10.1016/j.ypmed.2008.07.006. PubMed: 18675845. 18675845 10.1016/j.ypmed.2008.07.006

[10] Orrow G, Kinmonth A-L, Sanderson S, Sutton S (2012) Effectiveness of physical activity promotion based in primary care: systematic review and meta-analysis of randomised controlled trials. BMJ 344: e1389. doi:10.1136/bmj.e1389. PubMed: 22451477. 22451477 10.1136/bmj.e1389 PMC3312793

[11] Ronda G, Van Assema P, Brug J (2001) Stages of change, psychological factors and awareness of physical activity levels in the Netherlands. Health Promot Int 16: 305-314. doi:10.1093/heapro/16.4.305. PubMed: 11733449. 11733449 10.1093/heapro/16.4.305

[12] Lechner L, Bolman C, Van Dijke M (2006) Factors related to misperception of physical activity in The Netherlands and implications for health promotion programmes. Health Promot Int 21: 104-112. doi:10.1093/heapro/dal011. PubMed: 16641132. 16641132 10.1093/heapro/dal011

[13] van Sluijs EM, Griffin SJ, van Poppel MN (2007) A cross-sectional study of awareness of physical activity: associations with personal, behavioral and psychosocial factors. Int J Behav Nutr Phys Act 4: 53. doi:10.1186/1479-5868-4-53. PubMed: 17996060. 17996060 10.1186/1479-5868-4-53 PMC2186356

[14] Watkinson C, van Sluijs EMF, Sutton S, Hardeman W, Corder K et al. (2010) Overestimation of physical activity level is associated with lower BMI: a cross-sectional analysis. Int J Behav Nutr Phys Act 7: 68. doi:10.1186/1479-5868-7-68. PubMed: 20854659. 20854659 10.1186/1479-5868-7-68 PMC2954949

[15] DiClemente CC, Marinilli AS, Singh M, Bellino LE (2001) The Role of Feedback in the Process of Health Behavior Change. Am J Health Behav 25: 217-227. doi:10.5993/AJHB.25.3.8. PubMed: 11322620. 11322620 10.5993/ajhb.25.3.8

[16] Proper KI, Beek A, Hildebrandt VH, Twisk JW, van Mechelen W (2003) Short term effect of feedback on fitness and health measurements on self reported appraisal of the stage of change. Br J Sports Med 37: 529-534. doi:10.1136/bjsm.37.6.529. PubMed: 14665593. 14665593 10.1136/bjsm.37.6.529 PMC1724704

[17] McClure JB (2002) Are biomarkers useful treatment aids for promoting health behavior change?: An empirical review. Am J Prev Med 22: 200-207. doi:10.1016/S0749-3797(01)00425-1. PubMed: 11897465. 11897465 10.1016/s0749-3797(01)00425-1

[18] Bankhead CR, Brett J, Bukach C, Webster P, Stewart-Brown S et al. (2003) The impact of screening on future health-promoting behaviours and health beliefs: a systematic review. Health Technol Assess 7: 1-92. PubMed: 14670217. 10.3310/hta7420 14670217

[19] Kroeze W, Werkman A, Brug J (2006) A systematic review of randomized trials on the effectiveness of computer-tailored education on physical activity and dietary behaviors. Ann Behav Med 31: 205-223. doi:10.1207/s15324796abm3103_2. PubMed: 16700634. 16700634 10.1207/s15324796abm3103_2

[20] Smeets T, Brug J, de Vries H (2008) Effects of tailoring health messages on physical activity. Health Educ Res 23: 402-413. doi:10.1093/her/cyl101. PubMed: 17032705. 17032705 10.1093/her/cyl101

[21] Azjen I (1991) The theory of planned behaviour. Organ Behav Hum Decis Processes 50: 179-211. doi:10.1016/0749-5978(91)90020-T.

[22] Weinstein ND, Rothman AJ, Sutton SR (1998) Stage theories of health behavior: Conceptual and methodological issues. Health Psychol 17: 290-299. doi:10.1037/0278-6133.17.3.290. PubMed: 9619480. 9619480 10.1037//0278-6133.17.3.290

[23] Noar SM, Benac CN, Harris MS (2007) Does tailoring matter? Meta-analytic review of tailored print health behavior change interventions. Psychol Bull 133: 673–693. doi:10.1037/0033-2909.133.4.673. PubMed: 17592961. 17592961 10.1037/0033-2909.133.4.673

[24] Bravata DM, Smith-Spangler C, Sundaram V, Gienger AL, Lin N et al. (2007) Using pedometers to increase physical activity and improve health. JAMA 298: 2296-2304. doi:10.1001/jama.298.19.2296. PubMed: 18029834. 18029834 10.1001/jama.298.19.2296

[25] Shalowitz DI, Miller FG (2008) Communicating the Results of Clinical Research to Participants: Attitudes, Practices, and Future Directions. PLOS Med 5: e91. doi:10.1371/journal.pmed.0050091. PubMed: 18479180. 18479180 10.1371/journal.pmed.0050091 PMC2375946

[26] Gibbons FX, Gerrard M, Lane DJ, Mahler HI, Kulik JA (2005) Using UV photography to reduce use of tanning booths: a test of cognitive mediation. Health Psychol 24: 358-363. doi:10.1037/0278-6133.24.4.358. PubMed: 16045371. 16045371 10.1037/0278-6133.24.4.358

[27] Shahab L, Hall S, Marteau T (2007) The motivational impact of showing smokers with vascular disease images of their arteries: a pilot study. Br J Health Psychol 12: 275-283. doi:10.1348/135910706X109684. PubMed: 17456286. 17456286 10.1348/135910706X109684

[28] Kruger J, Blanck HM, Gillespie C (2006) Dietary and physical activity behaviors among adults successful at weight loss maintenance. Int J Behav Nutr Phys Act 3: 17. doi:10.1186/1479-5868-3-17. PubMed: 16854220. 16854220 10.1186/1479-5868-3-17 PMC1555605

[29] Nothwehr F, Yang J (2007) Goal setting frequency and the use of behavioral strategies related to diet and physical activity. Health Educ Res 22: 532-538. PubMed: 17032703. 17032703 10.1093/her/cyl117

[30] Watkinson C, van Sluijs EM, Sutton S, Marteau T, Griffin SJ (2010) Randomised controlled trial of the effects of physical activity feedback on awareness and behaviour in UK adults: the FAB study protocol. BMC Public Health 10: 144. doi:10.1186/1471-2458-10-144. PubMed: 20298560. 20298560 10.1186/1471-2458-10-144 PMC2859395

[31] De Lucia Rolfe E, Loos RJF, Druet C, Stolk RP, Ekelund U et al. (2010) Association between birth weight and visceral fat in adults. Am J Clin Nutr 92: 347–352. doi:10.3945/ajcn.2010.29247. PubMed: 20519560. 20519560 10.3945/ajcn.2010.29247

[32] Brage S, Brage N, Franks PW, Ekelund U, Wareham NJ (2005) Reliability and validity of the combined heart rate and movement sensor Actiheart. Eur J Clin Nutr 59: 561-570. doi:10.1038/sj.ejcn.1602118. PubMed: 15714212. 15714212 10.1038/sj.ejcn.1602118

[33] FAO/WHO/UNU (1985) Energy and protein requirements – Report of a joint expert consultation. Geneva: World Health Organization. 3937340

[34] Ainsworth BE, Haskell WL, Whitt MC, Irwin ML, Swartz AM et al. (2000) Compendium of physical activities: an update of activity codes and MET intensities. Med Sci Sports Exerc 32: S498-S504. doi:10.1097/00005768-200009001-00009. PubMed: 10993420. 10993420 10.1097/00005768-200009001-00009

[35] Brage S, Brage N, Franks PW, Ekelund U, Wong MY et al. (2004) Branched equation modeling of simultaneous accelerometry and heart rate monitoring improves estimate of directly measured physical activity energy expenditure. J Appl Physiol 96: 343–351. PubMed: 12972441. 12972441 10.1152/japplphysiol.00703.2003

[36] Thompson D, Batterham AM, Bock S, Robson C, Stokes K (2006) Assessment of low-to-moderate intensity physical activity thermogenesis in young adults using synchronized heart rate and accelerometry with branched-equation modeling. J Nutr 136: 1037–1042. PubMed: 16549471. 16549471 10.1093/jn/136.4.1037

[37] Strath SJ, Brage S, Ekelund U (2005) Integration of physiological and accelerometer data to improve physical activity assessment. Med Sci Sports Exerc 37: S563-S571. doi:10.1249/01.MSS.0000158181.75442.8B. PubMed: 16294119. 16294119 10.1249/01.mss.0000185650.68232.3f

[38] Besson H, Brage S, Jakes RW, Ekelund U, Wareham NJ (2010) Estimating physical activity energy expenditure, sedentary time, and physical activity intensity by self-report in adults. Am J Clin Nutr 91: 106-114. doi:10.3945/ajcn.2009.28432. PubMed: 19889820. 19889820 10.3945/ajcn.2009.28432

[39] Williams K, Prevost AT, Griffin S, Hardeman W, Hollingworth W et al. (2004) The ProActive trial protocol - a randomised controlled trial of the efficacy of a family-based, domiciliary intervention programme to increase physical activity among individuals at high risk of diabetes. BMC Public Health 4: 48. doi:10.1186/1471-2458-4-48. PubMed: 15491494. 15491494 10.1186/1471-2458-4-48 PMC526256

[40] Sutton S, French D, Hennings S, Mitchell J, Wareham N et al. (2003) Eliciting salient beliefs in research on the Theory of Planned Behaviour: The effect of question wording. Curr Psychol 22: 234-251. doi:10.1007/s12144-003-1019-1.

[41] Crockett RA, Weinman J, Hankins M, Marteau T (2009) Time orientation and health-related behaviour: Measurement in general population samples. Psychol Health 24: 333-350. doi:10.1080/08870440701813030. PubMed: 20204997. 20204997 10.1080/08870440701813030 PMC2657323

[42] Kinmonth A-L, Wareham NJ, Hardeman W, Sutton S, Prevost AT et al. (2008) Efficacy of a theory-based behavioural intervention to increase physical activity in an at-risk group in primary care (ProActive UK): a randomised trial. Lancet 371: 41-48. doi:10.1016/S0140-6736(08)60070-7. PubMed: 18177774. 18177774 10.1016/S0140-6736(08)60070-7

[43] StataCorp (2009) Stata Statistical Software, Release 11 College Station, TX: StataCorp LP.

[44] van Sluijs EMF, van Poppel MNM, Twisk JWR, van Mechelen W (2006) Physical activity measurements affected participants’ behavior in a randomized controlled trial. J Clin Epidemiol 59: 404-411. doi:10.1016/j.jclinepi.2005.08.016. PubMed: 16549263. 16549263 10.1016/j.jclinepi.2005.08.016

[45] King AC, Ahn DK, Oliveira BM, Atienza AA, Castro CM et al. (2008) Promoting physical activity through hand-held computer technology. Am J Prev Med 34: 138-142. doi:10.1016/j.amepre.2007.09.025. PubMed: 18201644. 18201644 10.1016/j.amepre.2007.09.025 PMC2715220

[46] McCrady-Spitzer SK, Levine JA (2010) Integrated electronic platforms for weight loss. Expert Rev Med Dev 7: 201–207. doi:10.1586/erd.09.73. PubMed: 20214426. 10.1586/erd.09.73 PMC2897161 20214426

[47] Sallis JF, Cervero RB, Ascher W, Henderson KA, Kraft MK et al. (2006) An ecological approach to creating active living communities. Annu Rev Public Health 27: 297-322. doi:10.1146/annurev.publhealth.27.021405.102100. PubMed: 16533119. 16533119 10.1146/annurev.publhealth.27.021405.102100

